# Development and Characterization of a Fucoidan-Based Drug Delivery System by Using Hydrophilic Anticancer Polysaccharides to Simultaneously Deliver Hydrophobic Anticancer Drugs

**DOI:** 10.3390/biom10070970

**Published:** 2020-06-28

**Authors:** Yen-Ho Lai, Chih-Sheng Chiang, Chin-Hao Hsu, Hung-Wei Cheng, San-Yuan Chen

**Affiliations:** 1 Department of Materials Science and Engineering, National Chiao Tung University, Hsinchu 30010, Taiwan; angus80817@gmail.com (Y.-H.L.); eddy610061@hotmail.com (C.-H.H.); mlb14756@gmail.com (H.-W.C.); 2 Cell Therapy Center, China Medical University Hospital, Taichung 40454, Taiwan; brian78630g@gmail.com; 3 Frontier Research Centre on Fundamental and Applied Sciences of Matters, National Tsing Hua University, Hsinchu 30013, Taiwan; 4 School of Dentistry, College of Dental Medicine, Kaohsiung Medical University, Kaohsiung ‎80708, Taiwan; 5 Graduate Institute of Biomedical Science, China Medical University, Taichung 40454, Taiwan

**Keywords:** fucoidan, PLGA, docetaxel, drug delivery system, anticancer therapy/cancer treatment

## Abstract

Fucoidan, a natural sulfated polysaccharide, which can activate the immune response and lessen adverse effects, is expected to be an adjuvant agent in combination with chemotherapy. Using natural hydrophilic anticancer polysaccharides to simultaneously encapsulate hydrophobic anticancer drugs is feasible, and a reduced side effect can be achieved to amplify the therapeutic efficacy. In this study, a novel type of fucoidan-PLGA nanocarrier (FPN-DTX) was developed for the encapsulation of the hydrophobic anticancer drug, docetaxel (DTX), as a drug delivery system. From the comparison between FPN-DTX and the PLGA particles without fucoidan (PLGA-DTX), FPNs–DTX with fucoidan were highly stable with smaller sizes and dispersed well without aggregations in an aqueous environment. The drug loading and release can be further modified by modulating relative ratios of Fucoidan (Fu) to PLGA. The (FPN 3-DTX) nanoparticles with a 10:3 ratio of Fu:PLGA displayed uniform particle size with higher encapsulation efficiency than PLGA NPs and sustained drug release ability. The biocompatible fucoidan-PLGA nanoparticles displayed low cytotoxicity without drug loading after incubation with MDA-MB-231 triple-negative breast cancer cells. Despite lower cellular uptake than that of PLGA-DTX due to a higher degree of negative zeta potential and hydrophilicity, FPN 3-DTX effectively exerted better anticancer ability, so FPN 3-DTX can serve as a competent drug delivery system.

## 1. Introduction

Cancer is one of the leading causes of death worldwide, and a major reason for the limited therapeutic efficacy against cancer is the difficulty to deliver therapeutic agents precisely and effectively to the tumor sites without inducing severe adverse effects on healthy tissues and organs [1,2]. Drug delivery systems (DDS) such as micelles, vesicles/liposomes, nanocapsules, porous particles, nanospheres, and inorganic nanoparticles have emerged to resolve therapeutic issues by enhancing the accumulation of active pharmaceutical molecules in tumor sites [3]. These nanosystems are capable of accumulating in tumors via passive targeting because leaky blood vessels in tumors induce enhanced permeability and retention (EPR) effects [4,5,6,7]. Among these DDSs, liposomes serve as prevalent carriers in biomedical applications, and several liposomal formulations have been clinically approved, e.g., Doxil^®^, Caelyx^®^, and Myocet^®^, for cancer treatment [8,9]. Hydrophilic compounds can be incorporated in the aqueous core of liposomes, whereas hydrophobic ones can be accommodated within the lipid bilayer of liposomes. Unfortunately, limited space offered by the lipid bilayer compromises the payload of hydrophobic compounds. Moreover, rapid release of hydrophobic drugs in the lipid bilayer will cause high drug leakage and limit the applicability of liposomes as an ideal candidate for DDS of hydrophobic drugs to the targeted sites [10,11,12]. On the other hand, Poly(lactic-co-glycolic acid) (PLGA) is one of the most successfully used biodegradable materials approved by the FDA for drug delivery due to its superior biocompatibility and biodegradability. PLGA with PEG surface modification (PEGylation) to increase the hydrophilicity of the formulation has been widely used to load and deliver a high payload of hydrophobic anticancer bioactive agents, including drugs, proteins/peptides, and small molecules [13,14,15,16,17]. 

In addition to common anticancer drugs, many natural polysaccharides acquired from natural sources such as plants and algae display anticancer properties. Fucoidan (Fu), a natural product from *Fucus vesiculosus*, has recently drawn considerable attention because of its anti-coagulant, anti-virus, and anti-thrombotic activities [18,19,20]. Pozharitskaya et al. further elucidates some mechanisms of radical scavenging and the anti-inflammatory, anti-hyperglycemic, and anti-coagulant bioactivities of high-molecular-weight fucoidan from *Fucus vesiculosus* using in vitro models [21]. Fucoidan can reduce cell proliferation, inhibit migration of cancer cells, and induce cell apoptosis. The anti-cancer effects and the bioavailability of fucodian are related to various fucoidan-mediated pathways including PI3K/AKT, the MAPK pathway, and the caspase pathway [22]. In addition, several case studies of fucoidan as an alternative medicine in animal and human clinical trials have proved that combining fucoidan with clinical therapeutic agents can alleviate side effects of anti-cancer chemotherapy [21,23]. Recently, Abdollah et al. [24] reported that fucoidan prolonged the circulation time of dextran-coated iron oxide nanoparticles (IONs) with a doubling in tumor uptake. Ikeguchi et al. [25] examined the synergistic effect of a high-molecular-weight fucoidan with colorectal cancer chemotherapy agents, oxaliplatin plus 5-fluorouracil/leucovorin (FOLFOX) or irinotecan plus 5-fluorouracil/leucovorin (FOLFIRI). In addition, it was reported that the degree of sulfation was one of the factors associated with the anticancer activity of fucoidan. Thus, highly sulfated fucoidans, mainly containing fucose residues, possess higher anticancer activities than heterofucans with a low degree of sulfation [26,27,28]. Fucoidan used in most anticancer studies is a commercially available and highly sulfated type extracted from *Fucus vesiculosus* [18]. The pharmacokinetic of fucoidan concentration was further evaluated using competitive ELISA or a more sensitive sandwich ELISA with fucoidan-specific antibodies (NCT03422055 and NCT0313082), which showed that the maximum concentration of fucodian was reached 4 hr after administration of a single dose in a rat model, and the relative bioavailability was very low [29]. Nagamine et al. demonstrated the uptake and distribution of 2% w/w dietary *Cladosiphon okamuranus* fucoidan in a rat mode [30]. The result showed that only 0.1% could be absorbed in Caco-2 cells. However, Kimura et al. [31] found that liposome NPs could improve the bioavailability of sulfated polysaccharide. Therefore, nanosystems or nanoparticles have been developed to promote the bioavailability of fucoidan. Thus, Fucoidan with PLGA was chosen in this study to develop nanoparticles as a drug delivery system. 

Docetaxel (DTX), used as a model drug in this study, has shown highly cytotoxic activity in several types of cancer including breast, lung, prostate, and ovarian cancers [32,33], but its clinical application is restricted owing to its poor aqueous solubility, low bioavailability, and cumulative systemic toxicity after prolonged and high-dose therapy [34]. Therefore, DTX is usually dissolved in Tween80: ethanol (50:50, v/v) to enhance its solubility, but these solvent-based DTX formulations easily cause toxic effects, including neutropenia, hypersensitivity, fluid retention, nail toxicities, and neuropathy. To enhance the bioavailability and anticancer activity, research has focused on entrapping DTX in nanocarriers such as polymeric micelles poly(lactic-co-glycolic acid) (PLGA) nanoparticles, and liposomes. Badran et al. reported that DTX loaded in chitosan(CS)-decorated PLGA NPs can maintain a higher concentration in the plasma with a longer terminal half-life and showed more than 4-fold the area under the plasma drug concentration-time curve (AUC) in CS-decorated PLGA NP compared to DTX solution [35]. Bowerman et al. [36] showed that DTX loaded in PLGA-nanoparticles can increase docetaxel circulation time. An in vivo antitumor efficacy study further demonstrated that DTX-NPs are expected to increase the therapeutic efficacy of chemotherapy and reduce systemic toxicity. Therefore, the DTX-encapsulated fucoidan-PLGA (FPNs–DTX) nanoparticles were developed to improve the therapy because fucoidan served as not only the anticancer agent but also one of the main components for stabilizing the nanoparticle structure. In addition, FPNs–DTX nanoparticles exhibit highly uniform particle size and excellent colloidal stability. As an inherently therapeutic nanomedicine with long-term circulation and high colloidal stability, FPNs–DTX are demonstrated to be potential candidate for cancer treatments.

## 2. Materials and Methods 

### 2.1. Materials

Fucoidan from *Fucus vesiculosus* (≥ 95%, Mw 20–200 kDa [37], 27.0% sulfate content [29], monosaccharides [38], Sigma, St. Louis, MO, USA), Resomer^®^ RG 502 H poly(D, L-lactide-co-glycolide) (PLGA, acid terminated, Mw = 7000–17,000), chloroform, acetonitrile (ACN, HPLC-grade), dialysis tubing cellulose membrane (molecular weight cut-off = 14,000), fetal bovine serum (FBS), bovine serum albumin (BSA), Trypsin-EDTA, paraformaldehyde, and dimethyl sulfoxide (DMSO) were purchased from the Sigma–Aldrich Chemical Co. (St Louis, MO, USA). Triton-X 100 was purchased from J.T. Baker. PELCO^®^ Center-Marked Grids (200 mesh, 3.0 mm O.D., Copper) were purchased from Ted Pella Inc. (Redding, CA, USA). CdSe/ZnS core/shell quantum dots (QDs, 620 nm and 520 nm) were purchased from Ocean Nanotech (Springdale, AR, USA). Docetaxel (≥ 99.5%) (DTX) was purchased from Scinopharm (Shan-Hua, Taiwan). 3-(4,5-dimethylthiazol-2-yl)-5-(3-carboxymethoxyphenyl)-2-(4-sulfophenyl)-2H- tetrazolium (MTS reagent) was purchased from Promega Co. (Madison, WI, USA). Alexa Fluor^®^ 488 Phalloidin and Gibco^®^ DMEM (Dulbecco’s Modified Eagle Medium) were purchased from Thermo Fisher Scientific (Waltham, MA, USA) and supplemented with 10% FBS (Gibco), 2 mM l-glutamine, penicillin/streptomycin (Sigma); Triple-negative breast cancer cells (MDA-MB-231) and Umbilical Cord-Derived Mesenchymal Stem Cells were kindly donated by Dr. Woei-Cherng Shyu (China Medical University, Taichung, Taiwan); 4’,6-diamidino-2-phenylindole (DAPI) was purchased from Invitrogen (Waltham, MA, USA). Fluorescence mounting medium was purchased from Dako (Agilent Technologies, Wood Dale, IL, USA). Phosphate-buffered saline (PBS) was purchased from AMRESCO (Solon, OH, USA). All chemicals and solvents were of analytical reagent grade.

### 2.2. Methods

#### 2.2.1. Preparation of Docetaxel-Encapsulated Fucoidan–PLGA Nanoparticles (FPNs–DTX)

Docetaxel (DTX) was used as a model drug to form FPNs–DTX and was useful to evaluate the drug loading and release behavior of these nanoparticles. The FPNs–DTX were fabricated by the emulsion and solvent evaporation method. The organic solvent (50 μL chloroform) comprising PLGA (3mg) and DTX (1 mg) was prepared and added to the fucoidan (0.5% *w*/*v*, 2 mL) aqueous solution. The mixed solution was subsequently emulsified by a probe ultrasonicator (UP200Ht-Handheld Ultrasonic Homogenizer, Hielscher Ultrasonics, Teltow, Germany) at the power of 10 W and the frequency of 26 kHz to form an oil-in-water emulsion. The organic solvent was then removed at 55 °C and the FPNs–DTX were washed with deionized water three times. Finally, the yield of FPN nanoparticles was about 18.4% (*w*/*w*). The abbreviations and compositions of all formulations used in this study are further summarized in Table 1.

#### 2.2.2. Characterization of FPNs–DTX

The average sizes and zeta potentials of the samples were measured by dynamic light scattering and electrophoretic light scattering, respectively (DLS and ELS, Delsa^TM^ Nano C analyzer, Beckman Coulter, Irving, TX, USA). The nanostructure of the FPNs–DTX was observed by field-emission scanning electron microscopy (FE-SEM, JSM-6700F, JEOL, Tokyo, Japan) and transmission electron microscopy (TEM, JSM-2100, JEOL, Japan). For SEM observation, the sample solution was dropped on silicon wafers, dried in a vacuum desiccator at room temperature, and coated with platinum sputtering. The element analysis was performed using energy-dispersive X-ray spectroscopy (EDS), with which SEM was equipped. For TEM observation, the sample solution was dropped on a carbon-coated copper grid. Residues of the droplet were removed, and then the copper grid was dried in a vacuum desiccator at room temperature. The chemical state of nanoparticles containing docetaxel was analyzed in the KBr mixture pellets using Fourier transform infrared spectroscopy (FTIR, spectrum 100, PerkinElmer, Waltham, USA).

For the X-ray photoelectron spectroscopy (XPS) detection, the sample-coated silicon wafers were prepared using the method similar to the SEM sample process. The chemical states of the specific element on the sample surface were determined with XPS at beamline 24A at NSRRC (National Synchrotron Radiation Research Center, Hsinchu, Taiwan). The XPS data were processed and peak-fitted using Origin software (OriginLab Corporation, Northampton, MA, USA).

The amount of fucoidan adsorbed onto the FPNs–DTX was evaluated by referring to the method in the study of Garti et al. [39]. In brief, after centrifugation of the FPNs–DTX emulsion for separation of the supernatant aqueous phase and the nanoparticles, the amount of excess fucoidan in the supernatant phase was lyophilized and then weighed. The amount of adsorbed fucoidan was estimated by the difference in weight of the preparation and the excess fucoidan in the supernatant phase.

#### 2.2.3. Drug-Loading Ability and In Vitro Drug Release

The loading capacity and encapsulation efficiency (EE) of FPNs–DTX were examined using high-performance liquid chromatography (HPLC). The sample solution was lyophilized and then weighed. The encapsulated DTX was extracted from nanoparticles with acetonitrile (ACN) and then quantified using HPLC (Agilent Technologies 1200 series, San Diego, CA, USA) with a 4.6 × 250 mm Inertsil^®^ ODS-2 (5 μm) column. The detector was operated at a wavelength of 227 nm, and the sample was eluted using a mobile phase composed of 40% deionized water and 60% ACN at a flow rate of 1.0 mL/min and ambient temperature. Peak identity was confirmed by the retention time of DTX of about 6.5 min. The loading capacity and EE of the DTX in the FPNs–DTX were calculated as follows:

Loading capacity% = A/B × 100%(1)

where A is the mass of encapsulated drug and B is the mass of all FPNs–DTX, while

EE% = C/D × 100%(2)

where C is the amount of encapsulated drug and D is the total amount of drug in the feeding formula.

For the drug release test, a mixture of FPNs–DTX (0.2 mL) suspension containing equivalent DTX and phosphate-buffered saline (PBS, 3.8 mL) in a volume ratio of 1:19 was added to a dialysis tubing cellulose membrane with a cutoff molecular weight of 14,000. The dialysis bags were dialyzed in phosphate-buffered saline (PBS, 10 mL) with 0.5% Tween 80 at 37 °C with gentle shaking, and aliquots of the incubation medium were collected at predetermined time points. The amounts of released drug were quantified using the HPLC method. The solution was maintained at a constant volume by replacing the original solution with PBS. All experiments were performed in triplicate.

#### 2.2.4. Comparison between PLGA Particles and FPNs

To study the role of fucoidan in forming a stable nanoemulsion, the appearance of the three emulsions containing fucoidan alone (Fucoidan), PLGA alone (PLGA), and fucoidan accompanying PLGA (Fu + PLGA) was observed and recorded over time after emulsification without removing the organic solvent. The amounts of ingredients and the preparation method were the same as those for FPNs–DTX prepared with PLGA (1 mg) but without DTX. For further comparing DTX-encapsulated PLGA particles (PLGA-DTX) with FPNs–DTX, characterizations were carried out by SEM, DLS, and ELS. The PLGA-DTX were fabricated according to the method of FPNs–DTX but in the absence of fucoidan.

To evaluate the stability of particles prepared with fucoidan (FPNs–DTX prepared with a fucoidan to PLGA ratio (10:3), abbreviated as FPN 3-DTX) or without fucoidan (PLGA-DTX corresponding to FPN 3-DTX, abbreviated as PLGA 3-DTX), particles were suspended in double distilled water (DDW) and fetal bovine serum (FBS). The particles were resuspended in DDW and FBS after centrifugation. Particle sizes were recorded over time by DLS measurement. The resuspended state of FPN 3-DTX versus PLGA 3-DTX in DDW after centrifugation and 10 min bath sonication was examined as well.

#### 2.2.5. Stability of FPN 3-DTX

To estimate the stability of FPN 3-DTX in terms of drug encapsulation and particle size, DTX concentrations and particle sizes were measured and recorded over time for four weeks by HPLC and DLS methods, respectively. The samples were stored at 4 °C. All experiments were performed in triplicate.

#### 2.2.6. Cell Culture

MDA-MB-231 breast cancer cells were seeded in 75T culture flasks containing Dulbecco’s Modified Eagle Medium (DMEM) supplemented with 10% FBS and 1% penicillin–streptomycin–neomycin solution at 37 °C under 5% CO_2_.

#### 2.2.7. Cellular Uptake and Subcellular Localization of the FPNs

To facilitate the observation of cellular uptake, hydrophobic red fluorescent QDs were added to the oil phase and then incorporated into FPNs prepared with a fucoidan to PLGA ratio (10:3) (FPN 3-QD) and PLGA particles corresponding to FPN 3-QD (PLGA 3-QD) according to the FPN 3-DTX fabrication process. For cellular association studied by flow cytometry, 2 × 10^5^ cells per well were seeded in 6-well plates and grown for 24 h. They were then incubated with nanoparticles for different periods of time. After incubation, cells were harvested by trypsin-EDTA and washed with PBS twice to remove free nanoparticles followed by analysis using a flow cytometer (NovoCyte^®®^, ACEA biosciences). The data were acquired from 10,000 cumulatively gated events (cells) by measuring their fluorescence intensity along with the number of events. 

In fluorescence microscopy and confocal laser scanning microscope (CLSM) experiments, cells were seeded on glass coverslips placed in well plates. After incubation, cells were incubated with nanoparticles for different periods of time. Subsequently, the cells were washed several times with PBS and fixed with 3.7% paraformaldehyde solution (in PBS) for 20 min. Cellular permeability was performed with 0.1% triton X-100 (in PBS) for 20 min. Blocking buffer (1% BSA in PBS) was added to the wells for 1 h. Finally, the cells were stained with blue-fluorescent 4’,6-diamidino-2-phenylindole (DAPI, 5 μg/mL) and green-fluorescent phalloidin (5 unit/mL) to visualize nuclei and F-actin, respectively, followed by mounting coverslips on glass slides using fluorescence mounting medium. Subcellular localization of FPN 3-QD and PLGA 3-QD was investigated using a fluorescence microscope (Eclipse TE2000-U, Nikon) and confocal laser scanning microscope (CLSM, D-Eclipse C1, Nikon).

#### 2.2.8. Cell Viability

In vitro cytotoxicity of free DTX, FPNs (FPN 3), PLGA particles (PLGA 3), DTX-loaded FPNs (FPN 3-DTX), and DTX-loaded PLGA particles (PLGA 3-DTX) was examined on MDA-MB-231 cells using the MTS method as follows: 2 × 10^3^ cells per well were first seeded in 96-well plates and exposed to serial concentrations of DTX or equivalent particles at 37 °C for 72 h. Subsequently, a 200 μL mixture of MTS reagent and FBS-free medium (1:5) replaced the original medium, and the cells were incubated for an additional 4 h. The absorbance was detected at a wavelength of 450 nm using a Sunrise microplate absorbance reader (Tecan, Austria). Finally, the cell viability was determined by comparison of sample absorbance (A _sample_) with the untreated control (A _control_) and calculated using the following equation:

Cell Viability (%) = (A _sample_/A _control_) × 100% (3)

The half maximal inhibitory concentration (IC_50_), which is defined as the dosage of a compound that inhibits 50% of cell growth, was calculated from the obtained viability curves using CompuSyn software (Version 1.0, 2004, ComboSyn Inc., Paramus, NJ). All experiments were performed at least in triplicate.

#### 2.2.9. Statistical Analysis

All means are presented with their standard deviation (SD). Analysis of variance (ANOVA) was conducted, and *p* < 0.05 was regarded as statistically significant.

## 3. Results

### 3.1. Preparation of Docetaxel-Encapsulated Fucoidan-PLGA Nanoparticles (FPNs–DTX)

**Scheme 1:**
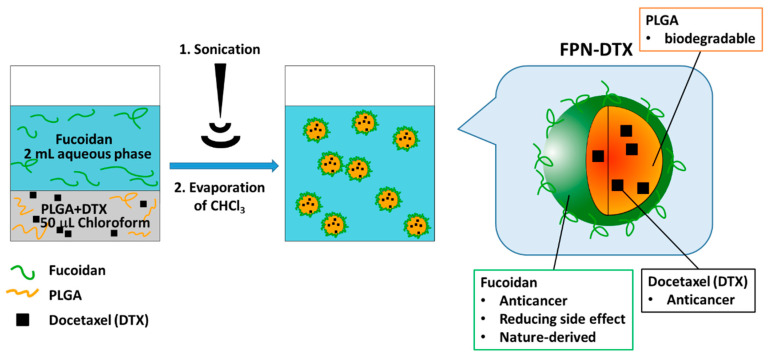
Illustration of FPNs–DTX preparation using a one-step emulsification process (Fucoidan in 2 mL aqueous phase and PLGA+DTX in 50 μL Chloroform phase).

Scheme 1 illustrates the fabrication process of the docetaxel-encapsulated fucoidan-PLGA nanoparticles (FPNs–DTX). To encapsulate the hydrophobic drug, various amounts of PLGA, which served as the main hydrophobic structure of the particle core, were adopted to form various FPNs–DTX. The formulas prepared with ratios 10:1, 10:3, and 10:5 (fucoidan to PLGA) were selected for further studies and are indicated as FPN 1-DTX, FPN 3-DTX, and FPN 5-DTX respectively. By mixing and emulsifying aqueous fucoidan solution in chloroform solution comprising PLGA and docetaxel, FPNs–DTX was obtained after removal of chloroform where the hydrophobic docetaxel drug was encapsulated within the PLGA matrix and fucoidan was exposed on the outside surface. To encapsulate the hydrophobic drug, various amounts of PLGA, which served as the main hydrophobic structure of particle core, were adopted to form various FPNs–DTX (Table 2). The encapsulation efficiencies (EE) and loading capacities for docetaxel showed that there was no positive correlation between encapsulation ability and PLGA content. As a result, the formulas prepared with ratios 10:1, 10:3, and 10:5 (fucoidan to PLGA) were selected for further studies and indicated as FPN 1-DTX, FPN 3-DTX and FPN 5-DTX, respectively.

### 3.2. Effects of Fucoidan on Nanoemulsions and Nanoparticles

**Figure 1:**
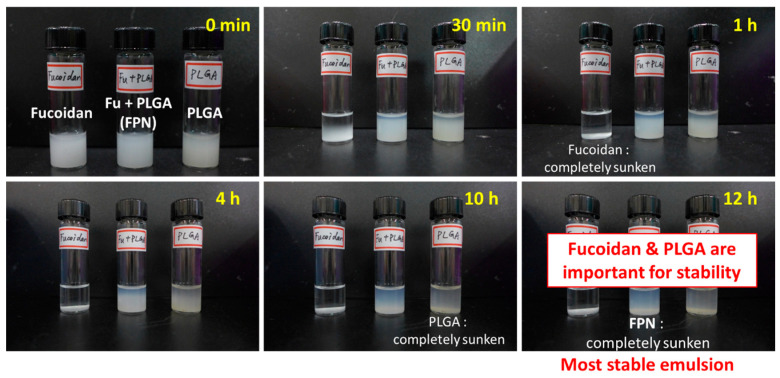
Observation of variation in emulsions containing fucoidan alone (Fucoidan), fucoidan accompanying PLGA (Fu + PLGA), and PLGA alone (PLGA) over time from the left to the right bottle in each image.

To study the effects of fucoidan on nanoemulsions, three emulsions containing fucoidan alone (Fucoidan), PLGA alone (PLGA), and a combination of fucoidan and PLGA (Fu + PLGA) were examined after emulsification without removing the organic solvent. By observing the variation of emulsions over time, it was found that white turbid layers in all three emulsions gradually sunk. The phase separation phenomenon called “creaming” or “sedimentation” is the migration of the dispersed phase in an emulsion as a reflection of instability. The sinking rate (sedimentation rate) can be an indicator of stability of the emulsions, while higher stability makes droplets sink more slowly [19,40]. In Figure 1, droplets with the highest to lowest sinking rate were ranked as fucoidan emulsion, PLGA emulsion, and Fu + PLGA emulsion. This indicated that the Fu + PLGA emulsion is the most stable among the three emulsions, implying the assistance of fucoidan in the stability of droplets or particles in the emulsion. However, it was also noted that the emulsion containing fucoidan alone was not sufficiently stabilized, so the hydrophobic material PLGA was necessary to form the rigid structure of the particles.

**Figure 2:**
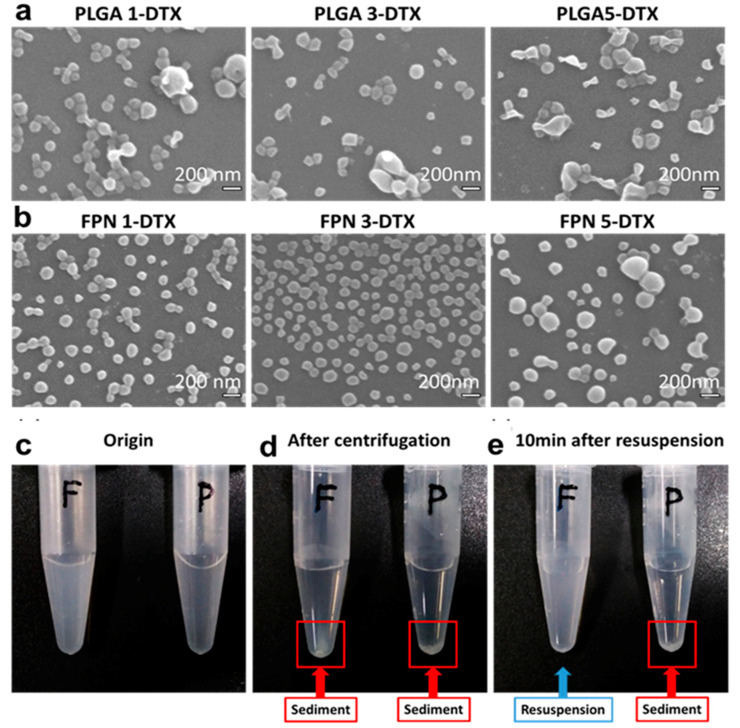
(**a**) SEM images of particles prepared without fucoidan (PLGA-DTX) compared with particles prepared with (**b)** fucoidan (FPNs–DTX). The concentration of PLGA in PLGA 1, PLGA 3, and PLGA 5 is equal to FPN 1, FPN 3, and FPN 5 respectively. Photograph of nanoparticles (FPN 3-DTX (F) and PLGA 3-DTX (P)) dispersive in DDW. In different states (**c**) the origins, (**d)** after centrifugation, and (**e**) 10 min after resuspension.

To further study the interaction between PLGA and Fucoidan in nanoparticles, SEM and TEM were used to observe the morphology of NPs. As illustrated in Figure 2a,b, the SEM image of particle morphology and distribution demonstrated the contribution of fucoidan to stabilize nanoparticles in suspension. Particles prepared without fucoidan (PLGA-DTX) did not exhibit uniform sizes and some of them aggregated together compared with those particles prepared with fucoidan (FPNs–DTX) as mentioned above. To investigate the surface hydrophobicity of particles prepared with or without fucoidan, the states of FPN 3-DTX (F) and PLGA 3-DTX (P) suspensions after centrifugation were observed in Figure 2d,e. Despite the similar appearance of both FPN 3-DTX and PLGA 3-DTX suspensions before and after centrifugation, FPN 3-DTX seemed to suspend and disperse well again in contrast to the sediment of PLGA 3-DTX for 10 min after resuspension. This indicates that the fucoidan of FPN 3-DTX can modify the surface hydrophobicity of PLGA and make particles disperse more easiily in an aqueous environment than PLGA 3-DTX, which is beneficial for delivery in the human circulatory system.

**Figure 3:**
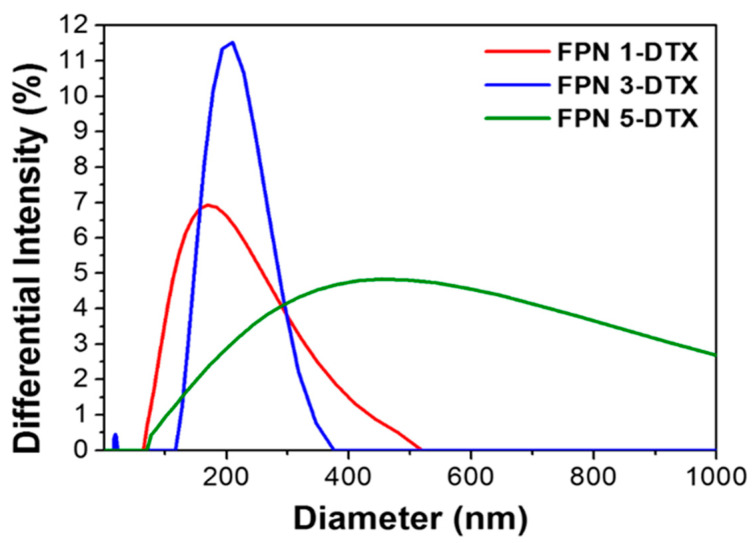
Size distributions of FPN 1-DTX, FPN 3-DTX, and FPN 5-DTX.

As observed from the DLS, particle size increased with an increasing amount of PLGA involved in preparation. FPN 3-DTX displayed the sharpest peak of size distribution and the smallest polydispersity index among the three formulas in Figure 3 based on DLS measurement, which is likely attributed to the negatively-charged fucoidan with Zeta potential (−60 mV) at the surface compared to those particles without fucoidan (PLGA-DTX = −25.2 mV), enhancing colloidal stability and the retention ability of FPNs–DTX in the human circulatory system.

### 3.3. Drug Release Behavior and Stability of FPNs–DTX

**Figure 4:**
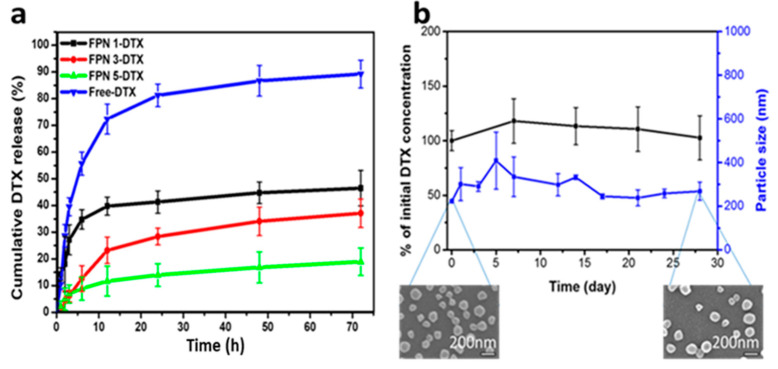
(**a**) Cumulative DTX release from FPN 1-DTX, FPN 3-DTX, FPN 5-DTX, and free DTX. (**b**) Variation in docetaxel concentration and particle size of FPN 3-DTX over time for four weeks.

To further study the release behavior of FPN nanoparticles, HPLC was used to detect the release of DTX from different ratios of FPN-DTX nanoparticles. Figure 4a shows in vitro natural drug release of FPNs–DTX in PBS. An initial burst release from FPN 1-DTX prepared with the least amount of PLGA was found, but the release exhibited a slower release rate as the amount of PLGA increased, which is possibly related to the change in the compactness of the particle structure. Therefore, FPN 3-DTX was selected for further evaluation because of three attractive features: (1) higher encapsulation ability for the drug, (2) excellent colloidal stability with uniform size, and (3) proper drug carrying ability rather than an initial burst release. To estimate the stability of FPN 3-DTX in suspension, particle sizes, morphologies, and the concentrations of drug encapsulation were measured and recorded over time for four weeks under 4 °C storage. The DTX concentration at various times was expressed as percentages of the initial concentration as shown in Figure 4b. Throughout this period, both indicators varied insignificantly (*p* > 0.05). The initial sample bore a great resemblance to the sample stored at 4 °C for about four weeks under SEM. In other words, FPN 3-DTX remained stable as a drug delivery system at least for about one month, which was probably attributed to the electrostatic interaction and steric hindrance from the nature of its anionic polysaccharide. Although fluctuations in FPN 3-DTX size were observed, the long-term stability of FPN 3-DTX was confirmed in Figure 4b.

### 3.4. Component Verification of FPN 3-DTX

**Figure 5:**
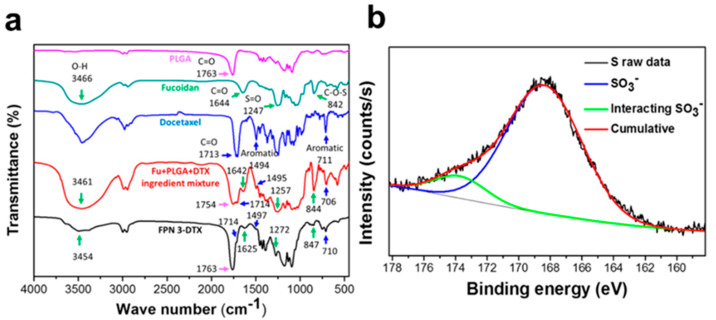
(**a**) Fourier transform infrared (FTIR) spectra of PLGA, fucoidan, docetaxel, powdered mixture of three main ingredients, and FPN 3-DTX. (**b**) Cumulative sulfur 2p (S 2p) X-ray photoelectron spectrum of FPN 3-DTX based on X-ray photoelectron spectroscopy (XPS).

The chemical bonds of PLGA, fucoidan, docetaxel, the Fu+PLGA+DTX mixed powder, and FPN 3-DTX were further measured (Figure 5a) using Fourier transform infrared spectra (FTIR). PLGA showed a major characteristic peak at 1763 cm^−1^ (COOH vibration). The spectrum of fucoidan presented a broad band at 3466 cm^−1^ (O-H groups stretching), a peak at 1644 cm^−1^ (COOH stretching vibration), a peak around 1247 cm^−1^ (S=O stretching), and a peak at 842 cm^−1^ (C-O-S bending). The major peak of docetaxel was around 1713 cm^−1^ (ester stretching). The spectrum of the powdered Fu+PLGA+DTX mixture showed major peaks of PLGA, fucoidan, and docetaxel even though some superpositions occurred. However, in the spectrum of docetaxel-encapsulated nanoparticles (FPN 3-DTX), the major peaks of docetaxel were significantly lowered, indicating the successful encapsulation of DTX drug in particles due to the chemical interaction between the carboxylic acid in PLGA and the ester group in docetaxel [41,42].

X-ray photoelectron spectroscopy (XPS) is a technique for identifying the composition of material surface and can be used to confirm the surface adsorption of fucoidan on nanoparticles. The XPS spectrum of the sulfur 2p (S2p) from FPN 3-DTX samples can be detected and decomposed into two bands by a curve fitting technique (Figure 5b). The binding energy peak around 168 eV represents sulfur at a higher oxidation state assigned to sulfur atoms bonded to oxygen atoms such as sulfone, sulfonate or sulfate [43,44]. In addition to the main peak around 168 eV, there was another one in a different position. The alteration of binding energy peak could indicate the interactions of some sulfate groups on fucoidan with the other components in FPN 3-DTX [45]. 

### 3.5. Cellular Uptake Efficiency and Subcellular Localization of Fluorescence-Labeled FPNs

**Figure 6:**
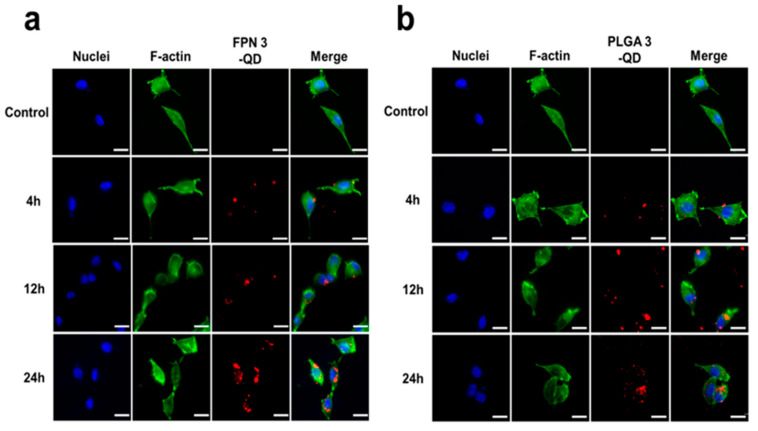
(**a**) Fluorescence images of cellular uptake of quantum dot-loaded FPN (FPN 3-QD) and (**b**) quantum dot-loaded PLGA particles (PLGA 3-QD) by MDA-MB-231 breast cancer cells at different times after feeding FPN 3-QD or PLGA 3-QD. Blue is DAPI for labeling the nuclei, green is phalloidin for labeling the cytoskeleton (F-actin), and red is the quantum dot from FPN 3-QD or PLGA 3-QD. (Scale bar: 20 μm).

One of the important concerns of cancer therapy is to deliver anticancer agents to the cancer cells in efficient and appropriate ways. Fluorescence microscopy images shown in Figure 6 demonstrated similar uptake behavior for FPN 3-QD and PLGA 3-QD. The QDs with the red fluorescence implied the subcellular localization of particles. Increasing red fluorescence intensity from both particles associated with cells was observed within 24 h incubation, when the amount of cell uptake seemed to reach saturation. 

**Figure 7:**
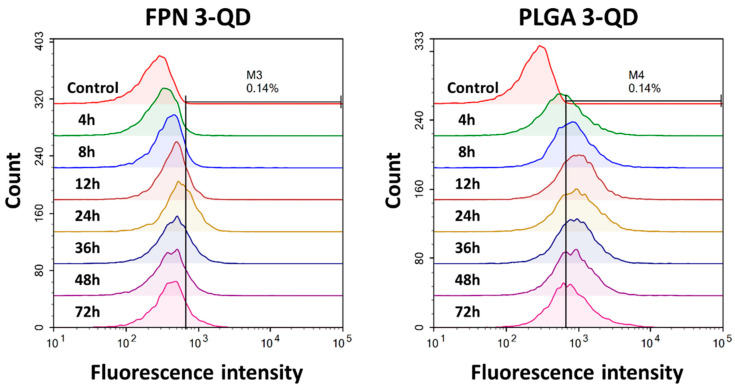
Flow cytometry for detecting cell associations with FPN 3-QD (F) and PLGA 3-QD (P) at different times after feeding particles. MFI in the tables stands for median fluorescence intensity. M3% (FPN 3-QD) and M4% (PLGA 3-QD) stand for the percent of the cell population emitting a fluorescence intensity in the range of the M3 gate and M4 gate, respectively.

Quantitative evaluation of cellular association was performed by flow cytometry as shown in Figure 7. The results were in line with the observations by fluorescence microscopy. The fluorescence intensity was observed to reach saturation at 24 h incubation for FPN 3-QD and at 12 h for PLGA 3-QD. The median fluorescence intensity (MFI) detected for PLGA 3-QD was higher than that for FPN 3-QD at all of the incubation times. On the other hand, higher fluorescence intensity compared with the untreated control indicates cell association with fluorescence-labeled particles. 

The behavior of cellular uptake assessed by fluorescence microscopy and flow cytometry indicated that the uptake of FPN 3-QD was inferior to that of PLGA 3-QD, which is possibly correlated with the higher degree of the negative zeta potential and hydrophilicity of FPN 3-DTX.

### 3.6. Cytotoxicity of FPN 3-DTX

**Figure 8:**
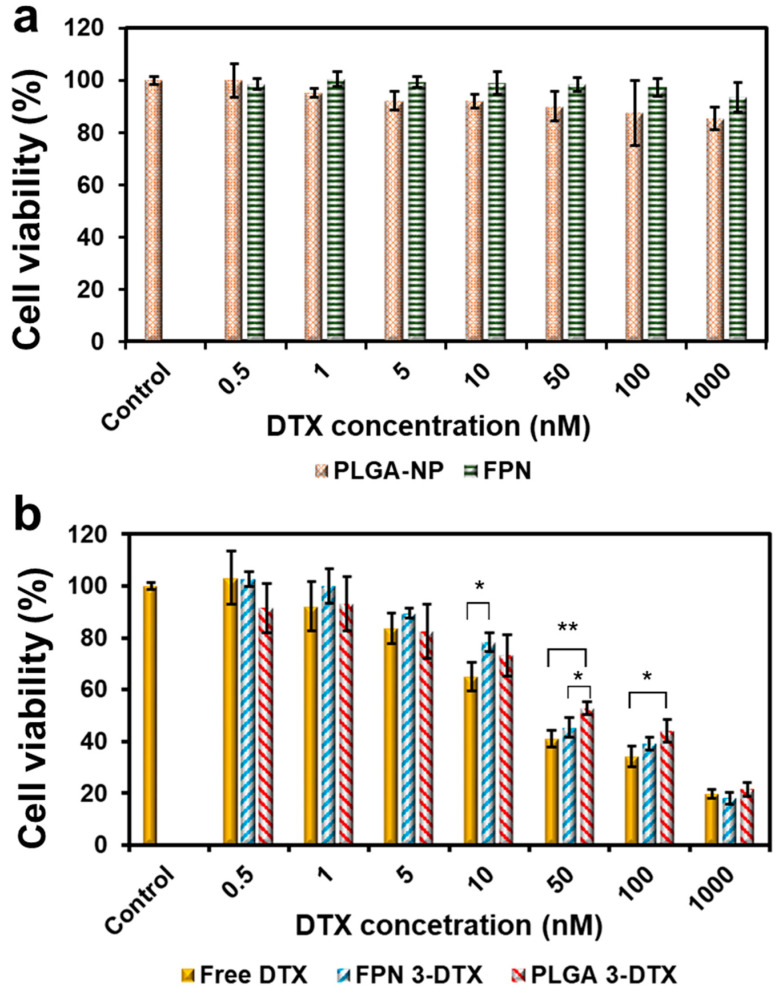
Cell viability of MDA-MB-231 breast cancer cells treated with (**a**) drug-loaded nanoparticles (FPN 3-DTX and PLGA 3-DTX) and free DTX. (**b**) blank nanoparticles (FPN and PLGA) in which the concentration of nanoparticles was equal to drug-loaded nanoparticles after 72hr treatment. Results are expressed as the mean ± SD of three replicates for each treatment (n = 3). Marked asterisks (*) with * *p* < 0.05 and ** *p* < 0.01 represent statistically significant differences.

The cell viability of FPN 3-DTX in a non-tumor cell line is very important and was first evaluated using umbilical cord-derived mesenchymal stem cells in this study. Figure S1 shows that FPN 3-DTX had higher cell viability compared to free DTX and blank nanoparticles (FPN) in the non-tumor cell line (mesenchymal stem cells (MSCs)) under the higher DTX concentration due to the protection of DTX within FPN. Subsequently, cytotoxicity of FPN 3-DTX was assessed in MDA-MB-231 breast cancer cells and compared with the other formulations as shown in Figure 8. Without DTX drug loading, it was found that FPN-3 displayed high biocompatibility in the absence of DTX compared to PLGA-3 (Figure 8a). With DTX loading, Figure 8b shows that the cell viability and IC_50_ indicated that FPN 3-DTX had a slightly lower cytotoxicity compared with PLGA 3-DTX at a very low dose because FPN 3-DTX possesses higher biocompatibility. However, FPN 3-DTX exhibited better cytotoxicity compared with PLGA 3-DTX as the DTX concentration was higher than 10nM. This suggested that by carefully assessing the therapeutic index, a proper dosage of FPN 3-DTX could be applied to achieve enhanced therapeutic efficacy using a lower DTX amount to reduce the side effect in comparison with free DTX and PLGA 3-DTX. 

## 4. Discussion

Fucoidan (Fu), a natural product from fucus vesiculosus, has recently drawn considerable attention because of its anti-coagulant, anti-virus, anti-thrombotic, and anticancer activities. Therefore, it was adopted as an excipient to produce FPN 3-DTX nanomedicine. The in vivo toxicity of the Fucodain-based drug delivery system was evaluated in our previous published paper [46] and demonstrated no obvious lesions in the liver, colon, lung or kidney based on histological analysis four weeks after the treatment of Fucoidan-base nanoparticles. Similar results were reported by Cai et al. when assessing the effect of PLGA with layer-by-layer (PLO/fucoidan) NPs on cell viability, acridine orange/ethidium bromide staining, hemolysis, and mouse systemic toxicity of cells and mice [47]. In addition, FPN 3-DTX with fucoidan modification on the surface of hydrophobic PLGA to encapsulate the hydrophobic drug DTX can produce more uniform and smaller particle sizes and also makes it easier for particles to disperse in an aqueous environment compared with PLGA 3-DTX, which is attributed to the higher hydrophilicity of FPN (Figure 2). The XPS result further confirmed the adsorption of fucoidan on the surface of FPN 3-DTX particles. In addition to interactions of the sulfate group, the formation of intermolecular hydrogen bonds could occur between the carbonyl groups of PLGA and the hydroxyl groups of fucoidan [48,49,50]. The advantage of using Fucoidan with PLGA addition can be further observed in Figure 4a which shows that FPN 3-DTX exhibited a slower release rate as the amount of PLGA increased, which is possibly related to the change in the compactness of the particle structure. Moreover, the FPN 3-DTX had high stability with hydrogen bonds which avoided the leakage of DTX and benefited long-time preservation as evidenced in Figure 4b. Barbosa et al. and Huang et al. further found that polysaccharide-base nanoparticles could improve the bioavailability [51,52]. Therefore, the FPN can be expected to improve the therapeutic effects with a high stability and low release rate. 

To visualize and quantify the cellular uptake of FPN3 for studying the delivery to cells, the quantum dots (QD) were introduced and encapsulated in the FPN3 to form the FPN 3-QD for fluorescence microscopy and flow cytometry using MDA-MB-231 breast cancer cells. We also tested QDs-loaded PLGA particles (PLGA 3-QD) to identify how zeta potential affects cellular uptake of particles. The behavior of cellular uptake assessed by fluorescence microscopy and flow cytometry indicated that the uptake of FPN 3-QD was inferior to that of PLGA 3-QD which is possibly correlated with the higher degree of negative zeta potential and hydrophilicity of FPN 3-DTX [53,54]. Without DTX drug loading, it was found that FPN-3 displayed high biocompatibility in the absence of DTX compared to the PLGA-3. The toxicity of FPN 3-DTX to normal cells is presented in Figure S1. The results showed that FPN had high biocompatibility and well protected DTX to prevent release from the NPs in the circulation system [55]. This indicated that FPN 3-DTX could reduce the side effect of DTX when transported to the tumor. These results were attributed to the slow release rate and lower cell uptake under the shorter incubation time (24h). Figure 8(b) further shows that FPN 3-DTX exhibited better cytotoxicity compared with PLGA 3-DTX as the DTX concentration was higher than 10nM. This indicated that FPN 3-DTX effectively exerted anticancer ability against MDA-MB-231 cells. In terms of better retention ability and tumor killing effect, FPNs–DTX can be regarded as a good potential drug delivery system (DDS) compared with PLGA 3-DTX. Moreover, the immune-related benefits of fucoidan such as activation of nature killer cells, an increase of IL-2 secretion, as well as inhibition of angiogenesis and metastasis will further enhance immunotherapy at a lower dose of the chemotherapy drug, which could not be observed and shown in vitro. Therefore, further experiments in vivo are necessary to evaluate the therapeutic efficacy of FPN 3-DTX.

## 5. Conclusions

In this study, a novel type of anticancer fucoidan-PLGA nanoparticle (FPNs–DTX) was developed that is capable of encapsulating hydrophobic docetaxel (DTX) as a drug delivery system. With the addition of PLGA, FPNs–DTX formed smaller nanoparticles and dispersed well without obvious aggregations over long-term duration. Furthermore, FPNs–DTX nanoparticles displayed low cytotoxicity and biocompatibility in the absence of docetaxel (FPN3) compared to PLGA. The more negatively charged and more hydrophilic properties of FPN 3-DTX could lead to lower cellular uptake but could effectively exert a better anticancer ability. Therefore, FPN 3-DTX is regarded as a better potential drug delivery system than PLGA 3-DTX in terms of better suspension and anti-cancer ability. The further achievements in vivo and the clinical applications of FPNs–DTX are promising in the biomaterial and cancer therapy fields.

## Figures and Tables

**Table 1:** Abbreviations and composition of all formulations.

Formulations	Fucoidan	Poly(lactic-co-glycolic acid)	Docetaxel	DTX-Loaded PLGA NPs	DTX-Loaded Fucoidan-PLGA NPs
Abbreviations	Fu	PLGA	DTX	PLGA 3-DTX	FPN 3-DTX
Composition	Fucoidan	PLGA	DTX	PLGA(3 mg) DTX(1 mg)	Fucoidan (10 mg) PLGA (3 mg) DTX (1 mg)

**Table 2:** Formulations and their corresponding encapsulated amounts, encapsulation efficiency (EE), and loading capacity of FPNs–DTX for docetaxel. Data in the table are the mean ± SD, n = 3.

Fucoidan:PLGA (*w*:*w*)	Encapsulated DTX (μg)	Encapsulation Efficiency (%)	Loading Capacity (%X)
10:1	551 ± 37	55.1 ± 3.7%	34 ± 2.3%
10:3	687 ± 42	68.7 ± 4.2%	28.7 ± 1.8%
10:5	611 ± 23	61.1 ± 2.3%	18.8 ± 0.7%
10:10	446 ± 16	44.6 ± 1.6%	6.6 ± 0.2%

## Supplementary Materials

The following are available online at https://www.mdpi.com/2218-273X/10/7/970/s1, Figure S1: Cell viability of umbilical cord-derived mesenchymal stem cells treated with free DTX, drug loaded nanoparticles (FPN 3-DTX) and blank nanoparticles (FPN).
