# Supplementary material for: Development and Characterization of a Fucoidan-Based Drug Delivery System by Using Hydrophilic Anticancer Polysaccharides to Simultaneously Deliver Hydrophobic Anticancer Drugs
Source: Biomolecules. 2020 Jun 28;10(7):970. doi: 10.3390/biom10070970 (PMC7408464; doi:10.3390/biom10070970)
Supplement: Supplementary file 1 [file biomolecules-10-00970-s001.pdf]

Supporting Information

For

Development and Characterization of Fucoidan-based Drug Delivery System by using hydrophilic anticancer polysaccharides to simultaneously deliver hydrophobic anticancer drugs

Yen-Ho Lai [1], Chih-Sheng Chiang [2], Chin-Hao Hsu [1], Hung-Wei Cheng[1] and San-Yuan Chen[1,3,4,5, *]

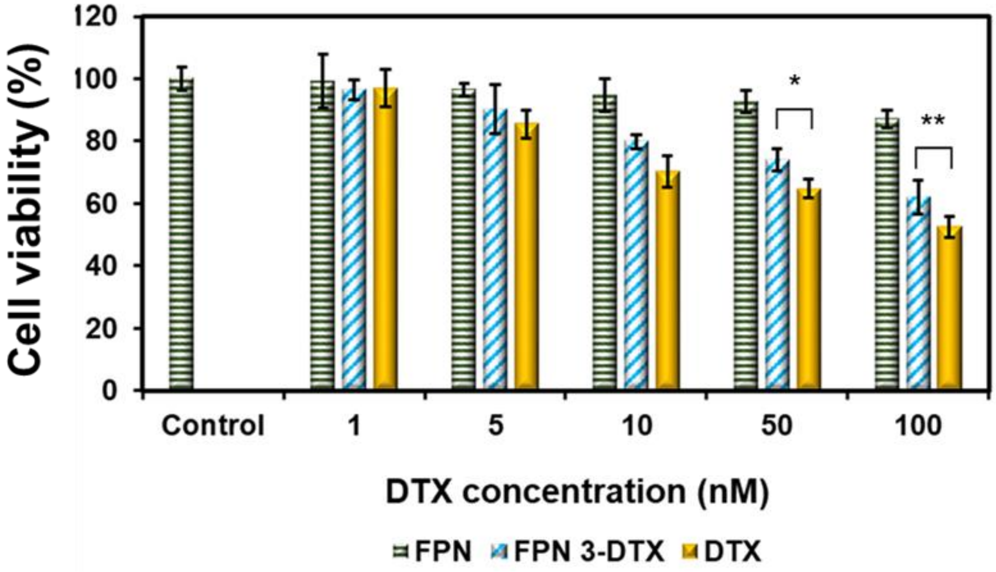

Figure S1. Cell viability of umbilical cord-derived mesenchymal stem cells treated with free DTX, drug loaded nanoparticles (FPN 3-DTX) and blank nanoparticles (FPN) in which concentration of nanoparticles are equal to drug loaded nanoparticles in mesenchymal stem cells (MSCs) after 24hr treatment. Results are expressed as the mean±SD of three replicates at each treatment (n = 3). Marked asterisks (*) with *$P < 0.05$ and **$P < 0.01$ representing statistically significant differences.
